# Treatment of Neuroendocrine Tumor Liver Metastases

**DOI:** 10.1155/2012/973946

**Published:** 2012-11-25

**Authors:** Mark A. Lewis, Timothy J. Hobday

**Affiliations:** Division of Medical Oncology, Mayo Clinic, Rochester, MN 55905, USA

## Abstract

In the care of patients with hepatic neuroendocrine metastases, medical oncologists should work in multidisciplinary fashion with surgeons, interventional radiologists, and radiation oncologists to assess the potential utility of liver-directed and systemic therapies. This paper addresses the various roles and evidence basis for cytoreductive surgery, thermal ablation (radiofrequency, microwave, and cryoablation), and embolization (bland embolization (HAE), chemoembolization (HACE), and radioembolization) as liver-directed therapies. Somatostatin analogues, cytotoxic chemotherapy, and the newer agents everolimus and suntinib are discussed as a means for controlling intra- and extrahepatic disease, along with peptide receptor radiotherapy (PRRT). Finally, the experience with orthotopic liver transplant for neuroendocrine tumors is described.

## 1. Introduction

The presence of hepatic metastases is the most important factor affecting the survival of patients with gastroenteropancreatic neuroendocrine tumors (GEP NETs) [1, 2]. Because of the portal venous drainage of the gastrointestinal tract and pancreas where most NETs arise, hematogenous spread to the liver is quite common, to the extent that dissemination from a primary GEP NET to the liver parenchyma will occur in at least 40% of patients [3], with some estimates ranging up to 85% [4]. Among these patients with hepatic metastases, about 75% are synchronous and evident at presentation, whereas 25% are metachronous and develop during the disease course [5]. The median overall survival in patients with hepatic metastases is 2–4 years [6, 7], and estimates for 5-year survival with untreated liver involvement range from 13 to 54% [8–10]. Beyond a shortened life expectancy, metastases can have a detrimental impact on patients' quality of life, especially through the carcinoid syndrome, in which vasoactive peptides that would normally be cleared by the enterohepatic circulation can cause profuse diarrhea, flushing, bronchospasm, damage to heart valves, and myriad other symptoms due to varied peptide hormone secretion. Often, metastatic involvement of the liver tends to occur well in excess of disease at extrahepatic sites. Understandably, there have been considerable efforts to limit the morbidity and mortality that patients incur from the metastatic burden of their NETs. The specialties of surgery, interventional radiology, and oncology all play a role in the multidisciplinary delivery of optimal care to these patients.

## 2. Surgery

Surgical resection of hepatic neuroendocrine metastases provides the greatest opportunity for long-term survival [11]. In patients with resectable liver lesions and with no extrahepatic disease beyond the primary NET, excision of the metastatic foci is often the only curative option. However, at the time hepatic metastases are first discovered, fewer than 20% of patients are eligible for metastasectomy or partial hepatectomy [12], either due to widely disseminated lesions or the anticipation that residual liver volume after resection will be functionally inadequate [4], so there is an inherent selectivity to the population whose outcomes are analyzed after these surgeries (Table 1).

The potential survival benefit of surgery has long been recognized. In 1992, Soreide et al. reported a retrospective cohort of 75 Norwegian patients with advanced carcinoid, 65 having midgut primary tumors and 18 exhibiting signs/symptoms of the carcinoid syndrome. Intra-abdominal debulking, not including liver resections, was performed in 33% of patients, with a median survival of 139 months in that operative group versus 69 months without debulking. The survival difference postoperatively was even more striking in the 48% of patients who underwent liver-directed interventions, versus those who did not: 216 months versus 48 months (*P* < .001), leading the authors to conclude that “the difference in survival probabilities in favor of aggressive surgical therapy is so marked that it is not unreasonable to conclude that surgery has played a role in prolonging life in these patients” [13].

A multi-institution review at 8 different hepatobiliary centers internationally examined clinical characteristics and outcomes in 339 patients undergoing resection of neuroendocrine liver metastases between 1985 and 2009. 60% of patients had bilateral liver involvement. 45% were treated with major hepatectomy, and 14% required staged operations with two separate procedures. 19% were treated with a combination of surgical resection and ablative techniques. Median survival was just over 10 years (125 months). Overall 5- and 10-year survival rates were 74% and 51%, respectively, though 94% of patients had developed new hepatic metastases within 5 years postoperatively. The greatest benefits were seen in patients with hormonally active NETs who had no macroscopically evident residual disease after surgery. In a multivariate analysis, a synchronous presentation, nonfunctional tumors, and extrahepatic disease were all statistically significant predictors of poorer survival [3].

A more recent systematic review by Saxena et al. of 29 studies conducted between 1990 and 2009 describing outcomes after hepatic neuroendocrine metastasectomy in a total of 1469 patients found a 63% median rate of R0 (microscopically negative) resection, a 5 cm median size of the largest excised tumor (ranging up to 9 cm), and a 95% rate of postoperative symptomatic relief. Median 5-year symptom-free survival was 37%. Progression-free survival (PFS) was reported in 12 of 29 studies, with a median PFS of 21 months, and median 5- and 10-year PFS rates of 29% and 1%, respectively. Overall survival (OS) was reported in 28 studies, but 11 had not reached median OS at the time of study publication; when calculable, the median OS was 70.5% at 5 years and 42% at 10 years [38]. The most common predictors of poorer survival in univariate analyses were macroscopically incomplete (R2) resections, extrahepatic disease, synchronous presentation, nonfunctional tumors, and poorly differentiated histopathology. The median perioperative mortality rate was 0%, and the median rate of surgical morbidity was 23%, with the most common complications including wound infections, intra-abdominal abscess formation, bile leak, and hepatic failure, none of which were seen at a median incidence exceeding 3%.

The discordance between PFS and OS in the above studies can be explained by the availability of effective postprogression treatments, including further liver-directed therapy and/or systemic approaches like somatostatin analogues [39], as well as the relative indolence of neuroendocrine tumors when compared to other malignancies that metastasize to the liver. However, because of the NETs' proclivity for recurrence, >90% debulking of the metastatic burden is recommended at the time of the initial cytoreductive surgery, to minimize the macroscopic or microscopic foci of disease that can then progress postoperatively. Less extensive debulking efforts seldom result in symptomatic or survival benefits for the NET patient [40]. In the Saxena et al. review, a median 5-year PFS of 21% and 5-year OS of 71.5% were seen in surgeries coupled to concurrent ablations, so favorable outcomes can be seen in patients who are undergoing procedures that are more elaborate than an isolated excision [38].

## 3. Ablation

During open or laparoscopic surgery, or during a dedicated image-guided percutaneous procedure, probes can be inserted which create either supraphysiologic heat or extreme cold, targeting the spherical area immediately around the instrument for destruction. Radiofrequency ablation (RFA) and microwave ablation (MWA) are the most frequently employed techniques to stimulate heat-related cell death [41]. Cryoablation, at the other end of the temperature spectrum, generates cytotoxic low temperatures and forms ice crystals out of intracellular water (Table 2). 

A large prospective study by Mazzaglia et al. of RFA, performed laparoscopically with ultrasound guidance, enrolled 54 patients with unresectable hepatic metastases from GEP NETs. Median survival after the first ablation was 3.9 years, although there was a bifurcation in the population between those patients whose largest metastasis exceeded 3 centimeters in size (median survival <3 years) and those whose dominant lesion was smaller than 3 centimeters (median survival not reached by study closure). Over 90% of patients reported postablation symptomatic improvement, and the median duration of symptom control was 11 months [25]. 

In 19 patients treated at their institution with 36 RFA procedures—all but one performed percutaneously—Gillams et al. observed, at a median followup of 21 months, a complete response (CR) in 6 patients, a partial response (PR) in 7, and stable disease (SD) in 1, controlling the hepatic disease burden in 14 (74%) of 19 patients. Nine (69%) of the 14 symptomatic patients achieved relief from hormone overproduction. There was 1 death from carcinoid crisis. The median postablation survival was 29 months [42].

Microwave ablation (MWA) may be more appropriate than RFA for targeting tumor sites next to major hepatic vasculature, where the adjacent blood flow theoretically predisposes RFA to a heat sink effect [41, 43]. The clinical experience with MWA has, to date, mostly involved treatment of hepatocellular carcinoma (HCC), but neuroendocrine tumors have been included in some series. Martin et al. described 11 NET patients undergoing MWA at their institution during a 5-year interval, for whom a 90% success rate for complete ablation was reported, with no recurrences observed at the ablation sites. The majority of these patients had MWA performed under ultrasound guidance during open surgery, that is, concomitant hepatectomy and/or extrahepatic metastasectomy. Median overall survival was 41 months [24]. There is still a paucity of data comparing MWA (especially performed percutaneously) to RFA, and geographic patterns of preference for one technique over the other are clear, with RFA more widely adopted in the United States and MWA more widely used in Europe and Asia [44].

In cryoablation, a sphere of ice forms around the probe, but intraprocedural temperatures will drop far below freezing point due to liquid nitrogen circulating in the metal instrument and, around −50°C, should induce necrosis in neoplastic tissue [45]. Seifert et al. described a series of 13 patients with NETs who underwent hepatic cryotherapy; under ultrasound, freezing continued until the ice extended 1 cm in each dimension around the tumor. 12 (92%) of 13 patients had complete ablation of all visible tumors, with 2 recurrences at the ablation sites and 12 survivors at 1 year of followup. All 7 patients who had hormonally related symptoms prior to cryotherapy experienced palliative benefit. Of note, 2 patients developed a postprocedural coagulopathy meriting factor replacement [27], and, in a larger series by Bilchik et al., all 17 patients undergoing hepatic cryotherapy for NETs developed a transient coagulopathy, requiring transfusion of either platelets or fresh frozen plasma (with an average infusion of 4 units per procedure) [46]. Animal models have confirmed that cryoablation results in a more exuberant inflammatory response and intravascular procoagulative/fibrinolytic state than heat-based thermal ablation techniques [47], and the severity of hematologic complications appears to correlate to the number of freeze-thaw cycles and the volume of frozen tissue [48].

## 4. Embolization

Healthy hepatocytes derive most of their blood supply from the portal vein, whereas neuroendocrine metastases are notable for their hypervascularity and reliance on the hepatic artery. These neoplastic attributes can be exploited both during diagnosis, where the tumors will be more conspicuous during the arterial phase of CT imaging, and during treatment, as the metastases are more vulnerable to necrosis if the hepatic artery is occluded. This selective vascular blockage can be accomplished through “bland” embolization (HAE), chemoembolization (HACE), or embolization with drug-eluting beads (DEB-HACE) (Table 3). In each method, vascular access is established via percutaneous catheterization of the femoral artery or, rarely, the brachial artery, and the cannula is then advanced into the relevant hepatic vascular territory. In bland embolization, an agent such as polyvinyl alcohol (PVA) is injected to impede blood flow in the engaged vessel until stasis is achieved, resulting in ischemia and then infarction in the downstream tissues. In chemoembolization, chemotherapeutic agents, most commonly doxorubicin, cisplatin, and mitomycin C, are mixed with an embolic agent, like ethiodized oil or lipiodol, and then the slurry is infused. 

To the best of our knowledge, there are no randomized studies directly comparing HAE to HACE. There has been an inconsistent suggestion of longer PFS and OS with HACE over HAE. Ruutiainen et al. found a 35% freedom from disease progression at 3 years in 44 patients undergoing HACE with cisplatin/doxorubicin/mitomycin C/iodized oil/PVA, versus 0% freedom from disease progression at 1 or 3 years in 23 patients undergoing HAE with PVA ± iodized oil; there was also a trend toward greater duration of symptomatic relief (15 versus 7.5 months) and high 5-year survival rates (50% versus 33%) [49]. In contrast, Pitt et al., in an analysis of 100 patients (49 HACE, 51 HAE), found similar rates of symptomatic improvement (88 versus 83%, resp.) and median OS (25.5 versus 25.7 months) [50]. 

Although intra-arterial chemotherapy may be expected to have more efficacy against islet cell tumors than midgut carcinoids [32], it is suspected that the ischemia-inducing component of the chemoembolization procedure is more therapeutically important than the antineoplastic effects of the accompanying chemotherapy, which may chiefly affect cells that would have been infarcted regardless. DEB-HACE aims for a more durable and less toxic impact from chemotherapy by loading larger embolic beads with a drug like doxorubicin that is then released slowly over 7–14 days, mainly into the hepatic parenchyma, with less systemic exposure and toxicity thereof. Bhagat et al. recently described an interim analysis of 13 patients enrolled in a phase II trial of DEB-HACE using 100–300 *μ*m beads loaded with ≤100 mg doxorubicin and up to 4 treatment sessions per patient within 6 months. At 1 month of followup, there was a mean 12% decrease in tumor size and 56% decrease in tumor enhancement, with an objective response rate of 78% by EASL (European Association for the Study of the Liver) criteria. At 6 months, there was a 90% disease control rate by RECIST criteria. The trial was interrupted, however, due to a higher-than-anticipated event rate for bilomas, in 7 (54%) of 13 patients (versus a 9.6% rate of biloma reported previously in the literature [51]). 4 of the patients with bilomas subsequently required percutaneous drainage, 3 for abscess formation and 1 for mass effect. The reopened trial's selection criteria now exclude patients whose largest lesion is <4 cm, and the drug-eluting beads are mixed in a 1 : 4 volume with contrast to maximize their visualization and delivery to the appropriate vascular territory [52]. 

These concerns about variable toxicities among the procedures notwithstanding, there is likely minimal clinical difference among HAE, HACE, and DEB-HACE in terms of efficacy, and the different techniques have similar exclusion criteria. All 3 approaches require that the patient has a patent portal vein to supply blood to the normal liver parenchyma; in complete portal vein thrombosis, iatrogenic embolization of the hepatic artery can result in fulminant hepatic failure. Patients whose synthetic liver function is also compromised by their NET metastases or other hepatic comorbidities are also at risk for decompensation with the inevitable embolic injury to noncancerous hepatocytes [53]. Partly because of this elevated risk, as well as historically lower response rates in patients with extensive liver involvement, >75% tumorous replacement of the hepatic parenchyma is a relative contraindication to embolization. 

The presence of unresectable extrahepatic disease should not be considered an absolute contraindication to pursuing liver-directed embolizations. Ho et al. reported the survival patterns of 46 patients (31 carcinoids, 15 islet cell NETs) following HAE or HACE, among whom there were 26 patients with appreciable extrahepatic disease. The survival time of patients without known extrahepatic metastasis (1571 ± 291 days) trended toward significance compared with those patients with distant disease (770 ± 112 days; *P* = .08) but the authors concluded that a postembolization survival benefit likely applied to all patients (having also observed no difference in survival between patients with resected and unresected primary tumors) and underscored an enhancement in quality of life too, insofar as there was an 80% rate of symptomatic improvement after the first embolization procedure [54].

It should be noted that traditional metrics of radiographic regression may not properly judge the success of any of the aforementioned embolic techniques. Under the RECIST criteria, central necrosis seen in a hypervascular neuroendocrine lesion following occlusion of its arterial blood supply may not be taken into account as a radiologic response if the cross-sectional area of the lesion remains unchanged [55], so many studies of liver-directed therapy in NETs have examined biochemical improvement, for example, decreasing chromogranin A, or amelioration of the carcinoid syndrome as therapeutic endpoints.

## 5. Radioembolization

Radioembolization offers another transarterial approach to unresectable neuroendocrine liver metastases. Yttrium-90 microspheres are a form of internal radiotherapy delivered to selected vascular territories, ideally with preferential engagement of vessels supplying the tumor and delivery of high radiation doses to these heavily perfused areas with relative sparing of normal liver tissue [56], which is often damaged in external beam radiation of the liver [30]. Theraspheres (MDS Nordion, Ottawa, ON, Canada) and SIR-Spheres (Sirtex Medical Limited, New South Wales, Australia) refer to proprietary radiopharmaceuticals that differ in the respective composition (nonbiodegradable glass *versus* biodegradable resin) and diameter (20–30 *μ*m *versus *20–60 *μ*m) of their microspheres [57]. 

A retrospective multi-institution review of radioembolization for neuroendocrine hepatic metastases was conducted by Kennedy et al., comprising 148 patients undergoing 185 separate procedures, all with resin SIR-spheres. The median radiation dose delivery was 1.14 GBq per procedure, and no radiation-induced liver failure was observed, even in 33 patients undergoing retreatment of the same hepatic lobe. Imaging response was evaluable by CT, MRI, or ^111^In-pentetreotide scintigraphy after 168 (91%) of 185 treatments, with 2.7% CR, 60.5% PR, and 22.7% SD. The median survival from the date of first microsphere treatment was 70 months; the vast majority of deaths were due to progression of metastatic disease in and outside the liver [30].

Proponents of radioembolization emphasize that, in general, fewer procedures and shorter hospital stays are needed than with HAE or HACE. In fact, in the Kennedy review, the preponderance of radioembolizations was performed on an outpatient basis. There have also been suggestions that there is lesser severity or incidence of postembolization pain [58, 59] compared to HA(C)E, although some of these observations have been made in the treatment of colorectal metastases [60] and HCC [61]. Another putative advantage is that repeated treatment may actually be more feasible in radioembolization due to a smaller “pruning” effect, that is, that the smaller embolic particles (35 *μ*m versus 100–300 *μ*m) leave more of the tumor's vascular supply patent if future embolizations are needed in the disease course [59]. 

Another important distinction from HAE and HACE is that radioembolization patients require preprocedural evaluation with technetium 99m (^99m^Tc)-labeled macroaggregated albumin (MAA) scans to rule out hepatopulmonary shunting [62] and avoid the life-threatening complication of progressive pulmonary insufficiency secondary to radiation pneumonitis [62]. Reflux of the microspheres into the gastroduodenal arteries can also cause radiation toxicity to the gut, potentially leading to severe ulceration that is poorly responsive to acid suppression [63]. 

There remains little complete long-term followup data regarding potential moderate radiation-induced hepatotoxicity with this approach, so some caution should be exercised for early use in patients that may live several years.

## 6. Peptide Receptor Radiotherapy (PRRT)

In patients whose NETs show at least liver-equivalent uptake of ^111^In-pentetreotide on scintigraphy, there is a therapeutic opportunity to deliver radioisotopes to the somatostatin-avid metastatic foci. The tissue penetration of ^111^In-pentetreotide itself appears poor, likely due to its small particle range, and early studies in the 1990s—at which time there were no other chelated somatostatin analogues labeled with *β*-emitting radionuclides [64]—demonstrated very low response rates, with reductions in tumor size seen in <10% of cases [65, 66], even at cumulative radiation doses exceeding 20 GBq, which were potentially myelosuppressive. More recently, other high-energy sources of *β*-emission, such as yttrium (^90^Y) and lutetium (^177^Lu), have been coupled to modified somatostatin analogues, for example, [Tyr^3^] octreotide, which have higher affinities for specific somatostatin receptors, as well as chelators, for example, tetraazacyclododecane tetraacetic acid (DOTA), which stabilize the binding [64]. These isotopes circulate throughout the body, and so they do not represent a liver-directed therapy inasmuch as they will bind to any somatostatin-avid focus of metastasis. In fact, this systemic distribution holds appeal in addressing the treatment of widespread inoperable disease, both intrahepatic and extrahepatic.

Kwekkeboom et al. described a large single institution experience with lutetium, in the form of ^177^[Lu-DOTA^0^,Tyr^3^]octreotate. Between 2000 and 2006, the Erasmus medical center administered 1772 PRRT treatments to 504 patients, all of whom were evaluable for early toxicity and 310 of whom were analyzed for efficacy. Patients received up to a cumulative radiation dose of 27.8–29.6 GBq (corresponding to a bone marrow exposure of ~2 Gy), usually in four treatment cycles, at treatment intervals of 6–10 weeks. Acute side effects included 25% nausea, 10% vomiting, and 10% abdominal pain, with 6 postprocedural hospitalizations for carcinoid crisis. WHO grade 3 or 4 hematologic toxicity occurred after at least 1 treatment in 10% of patients. Serious delayed toxicities included 4 cases of MDS, 2 episodes of renal failure, and 3 episodes of severe liver toxicity. Objective response rate, comprising CR, PR, and minor response (MR, tumor diameters decreasing between 25 and 50%), was 46%, with 2% CR and 30% PR. Gastrinomas, insulinomas, VIPomas, and nonfunctioning pancreatic NETs showed higher response rates than carcinoids. Three of four patients with previously inoperable pancreatic NETs responded to the extent that they could successfully undergo surgery. Median OS from initiation of PRRT was 46 months. The maximal effect of PRRT may take months after therapy to become evident in the shrinkage of metastases, because radiation damage to tumor DNA results in cell death only after one or more mitotic events, so Kwekkeboom et al. recommended the incorporation of PRRT early in the treatment plan of patients with extensive tumor load or hepatomegaly. Complete data on long-term toxicity is not available, as this is a clinical experience with variable followup, not clinical trial results. In addition, efforts to compare survival with this technique compared with older trials of various therapies are fraught with the expected cross-trial and cross-era biases.

These investigators also noted a longer residence time in tumors for [^177^Lu-DOTA^0^,Tyr^3^)]octreotate versus [^90^Y-DOTA^0^,TYR^3^]octreotide [67]. The same center had participated in a multi-institution phase I dose-escalation study of [^90^Y-DOTA^0^,TYR^3^]octreotide in 58 patients, 52 of whom had liver metastases, and saw 5 PR and 7 MR, for an ORR of 21%, and a median survival of 36.7 months [68]. These lower response rates and survival times with yttrium-based therapy contributed to their institutional preference for [^177^Lu-DOTA^0^,Tyr^3^)]octreotate as the somatostatin analog of choice for PRRT [67].

Of note, if progression is seen after the 1st cycle of PRRT, further treatments can be administered with a reasonable expectation of stabilizing disease. Pach et al. reported 16 patients with progressive disseminated NETs who received either lutetium-, yttrium-, or mixed lutetium/yttrium-octreotate, 10 of whom achieved SD at 6 months and 5 of whom maintained stability at 12 and 18 months, without an apparent significant increase in toxicity from repetition of radionuclide exposure [69]. 

## 7. Somatostatin Analogues

Analogues of somatostatin are used to control symptoms of hormonal overproduction by NETs, but they also exhibit an antiproliferative effect on tumor cells *in vitro* [70, 71]. In the largest *in vivo *study of this phenomenon to date, the PROMID study group conducted a placebo-controlled, double-blind, phase III trial of octreotide LAR in 90 patients with well-differentiated metastatic midgut NETs: 5 patients could not be randomized, 42 received depot octreotide 30 mg by intramuscular injection monthly, and 43 received placebo. 73 patients had liver metastases, 35 in the octreotide group (10 with >10% liver involvement and 17 with carcinoid syndrome) and 38 in the placebo group (11 with >10% liver involvement and 16 with carcinoid syndrome). The primary endpoint was time to progression (TTP). Median TTP in the recipients of octreotide LAR was 14.3 months (versus 6 months on placebo; HR = 0.34; 95% CI: 0.20–0.59, *P* = .000072). In a preplanned subgroup analysis, patients with >10% liver involvement appeared to receive less benefit from octreotide LAR in terms of TTP or tumor-related death, for example, 29.4 months on octreotide versus 6.1 months on placebo with 1–10% liver involvement, compared to 11.2 versus 5.5 months with 11–50% liver involvement [39].

The CLARINET trial is currently in progress as a randomized, double-blind, phase III study of lanreotide injections at 120 mg monthly doses in patients with nonfunctioning GEP NETs. By excluding patients with hormonal symptoms, the design of CLARINET is intended to focus as much as possible on the potential antiproliferative benefit of somatostatin analogs. At best, clinical stability and some control of endocrine syndromes are reasonable expectations of these therapies.

## 8. Chemotherapy

Historically, there has been tremendous variability in the reported response rates of NETs to systemic chemotherapy. These differences are likely explained by (1) different compositions of the regimens, for example, monotherapy versus doublets and triplets of cytotoxic agents, (2) different endpoints for response (assessed by radiologic, biochemical, and physical exam parameters or symptomatology), and (3) by disparities in underlying tumor biology. Theoretically, NETs with higher mitotic rates, reflected by higher Ki-67 labeling on pathology, may be more vulnerable to the antiproliferative effects of chemotherapy, and past studies did not uniformly account for this variable. Clinically, responses are much more likely in PNET than in midgut carcinoids.

In the early 1990s, Moertel et al. reported the efficacy of cisplatin/etoposide in anaplastic NETs with a closer histologic resemblance to small cell lung cancer than typical carcinoid or islet cell tumors. Among 27 patients with well-differentiated NETs (13 carcinoid, 14 islet cell), there were 2 PR, an ORR of 7%. Among 18 patients with anaplastic NETs, however, there were 9 PR and 3 CR, an ORR of 67%. The authors concluded that the histology was the primary determinant of response, as the two patient groups were otherwise comparable in terms of disease burden and performance status [72]. 

A 2004 retrospective study of streptozocin, 5-fluorouracil, and doxorubicin, in which 84 patients with locally advanced or metastatic pancreatic NETs received the triplet regimen irrespective of differentiation or mitotic rate, reported a 39% response rate; further subset analysis actually showed a longer PFS in low-grade tumors, but that the volume of hepatic metastases was the most influential predictor on outcome [73]. 

More recently, a 2007 study of capecitabine and oxaliplatin in 40 patients with advanced NETs found an ORR of 23% and biochemical response rate of 11% in 13 previously untreated poorly differentiated tumors, versus an ORR of 30% and 20% biochemical improvement in 27 patients with well-differentiated NETs progressing through somatostatin analogues [74]. Capecitabine was combined with the oral alkylator temozolomide in a retrospective review by Strosberg et al. including 30 chemotherapy-naïve patients with low-, intermediate-, or indeterminate-grade metastatic pancreatic NETs in whom the ORR was 70% [75]. Interestingly, a contemporaneous 2011 study by Welin et al. reported a 33% ORR to temozolomide-based therapy (± capecitabine ± bevacizumab) in 25 patients with NETs (17 with known GEP origin), all poorly differentiated or with a Ki-67 >20%, so alkylators may have efficacy against more unfavorable histologies as well [76]. 

Chemotherapy does not discriminate between intrahepatic and extrahepatic metastatic burden, complicating interpretation of its utility specifically for controlling neuroendocrine lesions in the liver. It should also be cautioned that some agents carry some cumulative risk of hepatotoxicity, for example, sinusoidal obstruction syndrome after oxaliplatin exposure [77]. 

## 9. Targeted Therapy

A more nuanced understanding of intratumoral pathways has led to the advent of targeted therapies for NETs that can be more discriminating than conventional cytotoxic chemotherapy. 

Angiogenesis plays a vital role in supporting neoplastic growth [78], and well-differentiated NETs in particular are rich in hypoxia-inducible factor (HIF) and vascular endothelial growth factor (VEGF), leading to a very high vascular density [79]. Liver metastases from well-differentiated pancreatic NETs show significantly upregulated VEGF-C expression, which may be involved in their progression [80]. Sunitinib is a multitarget tyrosine kinase inhibitor that affects VEGF receptors VEGFR-2 and VEGFR-2, as well as platelet-derived growth factor receptors (PDGFR *α* & *β*) [81], and stem-cell factor receptor (c-kit) [82]. On the basis of encouraging phase I and II trials in pancreatic NETs [83, 84], Raymond et al. conducted a randomized, double-blind, phase III trial of sunitinib versus placebo. 171 were enrolled, with 86 assigned to sunitinib and 85 to placebo. The trial was terminated early when 154 patients had undergone randomization. Median PFS was 11.4 months in the treatment group, versus 5.5 months in those on placebo. The ORR was 9.3% for sunitinib, versus no responses on placebo. The benefit for sunitinib was apparently lessened in subgroups where tumor Ki-67 exceeded 5% and where there was distant extrahepatic disease [85], but this remains exploratory. A recent single-institution phase II trial examined the role of sunitinib administered between serial HAEs to determine if a systemic antiangiogenic agent could augment the localized devascularization accomplished through embolization. Thirty-nine patients underwent a median of 2 HAEs, with sunitinib given at a starting dose of 37.5–50 mg beginning a week after the 1st embolization, up to a maximum of 8 six-week (four weeks on-therapy/two weeks off-therapy) cycles. Sixteen patients required dose reductions to 25 mg due to side effects, for example, nausea/vomiting, diarrhea, and poorly controlled hypertension. This study suggests that sunitinib is difficult to tolerate after HAE. The authors commented that the 66% rate of PFS at 1 year and the 59% rate of OS at 4 years improved upon the published retrospective experience with HAE, but definitive conclusions cannot be made regarding efficacy as outcomes from embolization alone vary widely among single institutions [86].

Everolimus inhibits mammalian target of rapamycin (mTOR), a serine-threonine kinase that promotes downstream overexpression of several growth factors and their receptors in NETs [87]. Inhibition of mTOR has an established antiproliferative effect on pancreatic NETs [88, 89], which led Yao et al. to perform a randomized, phase III study of everolimus versus placebo in 410 patients with advanced, low- or intermediate-grade pancreatic NETs progressing within the preceding 12 months; patients who progressed during the study while on placebo were allowed to crossover to open-label everolimus. 207 patients were randomized to everolimus, and 203 to placebo. The median PFS in an intention-to-treat analysis was 11.0 months on everolimus, versus 4.6 months on placebo, with an HR for disease progression or all-cause mortality on everolimus of 0.35, 95% CI: 0.27–0.45, *P* < .001. A prespecified subgroup analysis showed that the everolimus benefit persisted irrespective of prior chemotherapy, prior somatostatin analog use, or tumor grade. 92% of the patients enrolled had liver metastases, and were evenly distributed between the treatment and placebo arms, so this population comprised most of the cohort and was thus not deemed a subgroup. A difference in OS was not seen, but there was 73% crossover from placebo to open-label everolimus which may have confounded a potential treatment-related survival advantage [90]. In a similar randomized trial in 429 patients with progressive functioning carcinoid tumors, an absolute improvement in PFS was demonstrated (16.4 months versus 11.3 months, HR 0.77, 95% CI 0.59–1.00, *P* = .026). Although CIs include 1.00, a careful review of the trial suggests clinical utility in our opinion [91]. 

Again, questions have been raised about the appropriate sequencing of targeted therapies among other systemic approaches and liver-directed interventions. We feel targeted therapy is appropriate for patients with progressive liver metastases with modest symptoms where tumor stability would yield a clinical benefit. The randomized trials have not carefully documented expected degress of improvement in hormonal symptoms for functioning tumors. If symptoms are difficult due to disease bulk in the liver or extensive hormonal symptoms not controlled with somatostatin analogues, liver directed therapies, PRRT or chemotherapy (in PNET) may be preferred.

## 10. Liver Transplant

The United Network of Organ Sharing (UNOS) oversees the distribution of donated organs in the United States, and a 2011 retrospective analysis of the UNOS database by Gedaly et al. identified that, among 87820 liver transplants performed between 1998 and 2008, 150 orthotopic liver transplants (OLT) were for metastatic NETs, with 51 (34%) carcinoids, 29 (19%) hormonally active pancreatic NETs, and 70 (47%) unspecified NETs. Median age was 45 years. 13 patients received another organ at the time of OLT. Overall survival at 1 year was 81%, versus 65% and 49% at 3 and 5 years, respectively. There was no statistically significant difference in outcome between carcinoid and islet cell tumors. The median wait time for a donor organ was 67 days, and the authors actually identified an improved long-term survival in patients with above-median wait time than below-median wait time (63% versus 36% at 5 years, *P* = .005); a shorter wait time was particularly associated with shorter survival in patients older than 55 (17% 5-year survival, versus 41% 5-year survival in patients ≤55 years old). If wait time was excluded from analysis, then univariate consideration of age did not have a statistically meaningful impact on survival. Of the 83 patients with available recurrence data, 77% were alive without recurrence at 1 year, versus 50% at 3 years, and 32% at 5 years. The study authors concluded that survival in OLT for NETs was comparable to that seen in the much more common practice of OLT for HCC, although the NET recurrence rate of 31% was roughly double the historical observations of 10–15% posttransplant recurrence of HCC [34]. 

Similar to the survivals observed in the Gedaly analysis, a meta-analysis by Mathe et al. of 20 studies encompassing 89 NET patients (69 with pancreatic NETs, 61 of which were functional) undergoing OLT reported cumulative 1-, 3-, and 5-year survival rates of 71%, 55%, and 44%, respectively. Recurrence-free survivals were 84%, 47%, and 47% at the same time points. In patients ≤55 years old not undergoing simultaneous pancreatic resection, the predicted 5-year survival was 61%. In patients >55 undergoing resection of the primary pancreatic lesion at the same time as OLT, there was a 0% 5-year survival. Accordingly, the authors recommended that patient selection for transplant account for age and simultaneous extrahepatic resections [35]. This finding was corroborated in a multicenter French study by Le Treut et al. in 85 cases of OLT for NETs, 34 of the patients underwent concurrent resection of extrahepatic disease, and 7 of whom required upper abdominal exenteration (resection of the pancreas, spleen, stomach, and duodenum, with 3 patients receiving *en bloc *composite liver-duodenum-pancreas grafts). Concurrent exenteration had the strongest association with death in multivariate analysis (RR: 3.72, 95% CI: 1.54–8.95, *P* = .0034), with 0% 3-year survival and a median survival of 1.5 months, compared to a median OS of 56 months in the entire cohort [36]. Rosenau et al. added pathologic factors as important predictors of long-term survival, finding, among 19 NET patients undergoing OLT, that survival in the 5 patients with low Ki-67 and regular E-cadherin staining was significantly superior to the 12 patients with high Ki-67 or aberrant E-cadherin expression (7-year survival 100% versus 0%, *P* = .007) [37].

While OLT for NET remains controversial given the limited supply of donor organs, and a relatively high rate of recurrent disease is a legitimate concern, it is clear from these studies that many clinicopathologic variables, including surgical plans for concurrent resection of extrahepatic disease, have to be taken into careful account before pursuing transplantation (Table 4). We have offered OLT to select patients with diffuse hepatic involvement not amenable to standard hepatic debulking, no extrahepatic metastatic disease, resected or resectable primary tumors, and otherwise excellent health. Younger patients with difficult syndromes caused by peptide hormone secretion may merit even stronger consideration.

## 11. Summary

In the care of patients with hepatic neuroendocrine metastases, medical oncologists should work in multidisciplinary fashion with surgeons, interventional radiologists, and nuclear medicine physicians to assess the potential utility of liver-directed and systemic therapies. While the optimal sequence of many ablative, embolic, and pharmacologic interventions remains unclear, it is certain that the first step in management should be an assessment of patient eligibility for hepatic metastasectomy, which is associated with the best long-term outcomes. Thereafter, the lack of a clear evidence basis for the order of interventions may actually provide the practitioner with more flexibility and a greater number of attempts at establishing disease control while maintaining quality of life. Somatostatin analogues can be used for both symptomatic relief from hormonal excess and for their antiproliferative effect. At the time of this writing, PRRT is not yet widely available in the United States, but diagnostic scintigraphy can be helpful in identifying extrahepatic disease and focusing efforts at disease control inside and outside the liver. For patients with symptoms of bulky hepatic disease or from functional tumors, liver-directed therapies may be appropriate. The targeted therapies everolimus and sunitinib could be considered for use before or after conventional chemotherapy. Orthotopic liver transplantation may be pursued in special circumstances, but is not considered standard treatment.

## Figures and Tables

**Table 1:** Summary of outcome from resection of neuroendocrine liver metastases.

First author, publication year	Number of surgical patients	Median followup, months	Survival data	Predictors of survival
Mayo, 2011 [14]	339 (66 with simultaneous ablation)	26	Median OS: 123 months 5-year survival: 74%	High-volume (>25% liver involved) and symptomatic disease benefited most from surgery (versus intra-arterial therapy, *P* < .001)

Saxena, 2011 [15]	74 (38 with simultaneous cryoablation)	41	Median PFS: 23 months Median OS: 95 months	PFS: pathologic margin status (*P* = .023) OS: grade (*P* < .001), extrahepatic disease (*P* = .021)

Karabulut, 2011 [16]	27	29	Median PFS: 15 months Median OS: 190 months	Improved OS with resection of primary tumor (*P* = .01)

Glazer, 2010 [17]	172 (120 with small bowel or pancreatic primaries; 18 had only RFA)	50	Median OS: 116 months 5-year survival: 77.4% 10-year survival: 50.4%	Increasing interval from primary resection to hepatic metastases predicted for poorer survival (*P* = .01)

Fischer, 2008 [18]	118	20	5-year survival: 44% for well-differentiated neuroendocrine carcinoma versus 0% for poorly-differentiated	In well-differentiated carcinomas, any resection (R0 versus R1/2) significantly increased survival (*P* = .003)

Osborne, 2006 [19]	70		Mean OS: 50 months for complete cytoreduction (versus 32 months for palliative cytoreduction)	

Sarmiento, 2003 [20]	170 (75 with complete resection)		Median OS: 81 months	

Elias, 2003 [21]	47 (36 with concurrent extrahepatic resection)	62	Median OS: 91 months 5-year survival: 71%	DFS: completeness of surgery (R0 versus R1 versus R2) (*P* = .003), pancreatic origin (*P* = .01), bilateral liver involvement (*P* = .01)

Chen, 1998 [22]	15		5-year survival: 73% (versus 29% in 23 patients with unresectable disease)	

**Table 2:** Summary of outcomes for ablation of neuroendocrine liver metastases.

Author, publication year	Number of ablated patients	Median followup, months	Survival data	Comments
Karabulut et al., 2011 [16]	69 (RFA)	22	Median PFS: 10.5 months Median OS: 73 months	No significant overall survival difference between RFA and resection

Akyildiz et al., 2010 [23]	89 (RFA; 78 with NETs of GI origin, 11 medullary thyroid cancer)	30	Median DFS: 15.6 months Median OS: 72 months	Liver tumor volume (>76 cc versus <30 cc, *P* = .04), symptoms (present versus absent, *P* = .04), extrahepatic disease (present versus absent, *P* = .02)

Martin et al., 2010 [24]	11 (MWA; 7 with concomitant hepatectomy; 6 with concomitant extrahepatic resection)	36	Median DFS: 8 months Median OS: 18 months	Zero recurrences at ablation site

Mazzaglia et al., 2007 [25]	63 (RFA; 24 with extrahepatic disease at time of 1st ablation)	34	Median OS: 47 months after 1st RFA 5-year survival: 48%	Male gender (3x mortality risk of female) (*P* = .04), largest tumor > 3 cm (*P* = .03)

Gillams and Lees, 2005 [26]	25 (RFA)	21 (in 19 patients)	Median OS: 29 months	Shorter survival (23 months) in carcinoid patients

Seifert et al., 1998 [27]	13 (cryoablation)	13.5	12 patients alive at the end of followup (up to 103 months)	All 7 symptomatic patients had subjective improvement

Shapiro et al., 1998 [28]	5 (cryoablation)	30	1-year survival: 60% 2-year survival: 40%	All 5 patients had relief of carcinoid syndrome

**Table 3:** Summary of outcomes for intra-arterial therapy of neuroendocrine liver metastases.

First author, publication year	Number of embolized patients	Survival data	Comments
Paprottka, 2011 [29]	42 (^90^Y radioembolization)	40 of 42 patients alive with mean followup of 16.2 months	No radiation-induced liver failure; 36 of 38 symptomatic patients improved clinically within 3 months

Kennedy, 2008 [30]	148 (^90^Y radioembolization)	Median OS: 70 months	No radiation-induced liver failure

Strosberg, 2006 [31]	84 (HAE)	Median OS: 36 months	Fewer symptoms in 44 of 55 symptomatic patients

Gupta, 2005 [32]	123 (74 HAE, 49 HACE)	Median OS (carcinoid): 33.8 months Median OS (islet cell): 23.2 months	Male gender (versus female) predicted worse OS (*P* = .05) for carcinoid, bone mets predicted worse OS for islet cell (*P* = .03)

Dong, 2011 [33]	123 (HACE)	Mean OS: 39.6 months 5-year OS: 36% 10-year OS: 20%	Baseline albumin <3.5 g/dL was multivariate predictor for poorer OS (*P* = .003)

**Table 4:** Summary of outcomes for liver transplantation for neuroendocrine metastases.

Author, publication year	Number of liver transplant (LT) patients	Survival data	Predictors of survival
Gedaly et al., 2011 [34]	150 (13 receiving another organ at time of LT)	49% 5-year survival	Improved survival with patients waiting more than 2 months for transplant (*P* = .005), esp. in patients >55 years old

Mathe et al., 2011 [35]	89	44% 5-year survival	0% survival if >55 years old undergoing simultaneous pancreatic resection

Le Treut et al., 2008 [36]	85 (34 with concurrent extrahepatic resection)	Median OS: 56 months	Exenteration, duodeno-pancreatic primary, and hepatomegaly were indicators of poor prognosis (all RR of death > 2.6)

Rosenau et al., 2002 [37]	19	50% 10-year survival	Ki-67 <5% and normal E-cadherin expression had 100% 7-year survival
